# Supplementary figures and images for: Dual-purpose dynamics emerge from a heterogeneous cell population in Drosophila metamorphosis
Source: PLoS Comput Biol. 2025 Aug 28;21(8):e1013331. doi: 10.1371/journal.pcbi.1013331 (PMC12393715; doi:10.1371/journal.pcbi.1013331)

**S1 Fig**

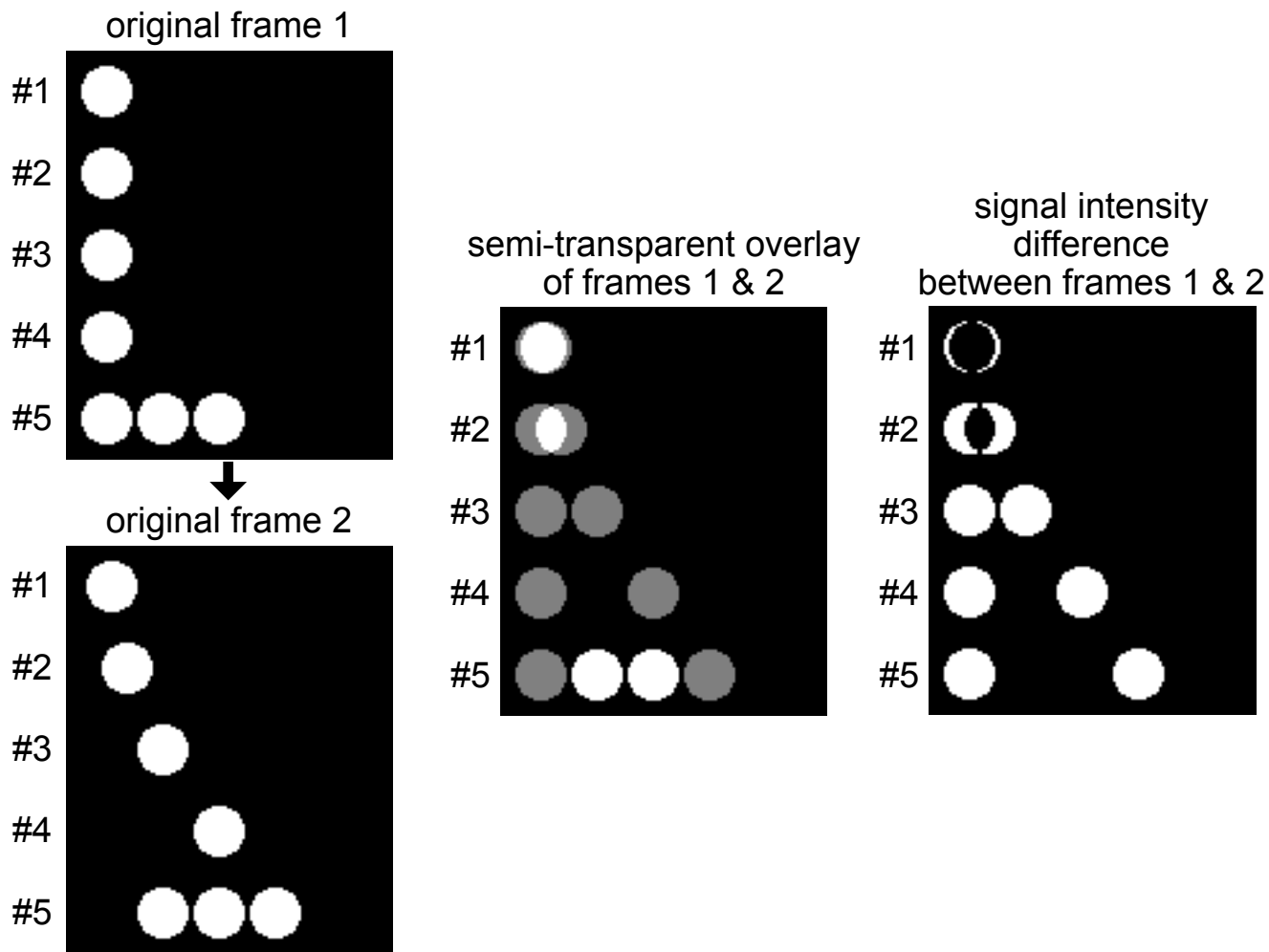

Supplement: S1 Fig — Let original images consist of two frames (left), from which one signal intensity difference image are obtained (right). The middle shows the semi-transparent overlay of the two original images to help understanding. Assume white particles are moving rightward with different speeds. The slower a particle moves, the smaller the area of signal intensity-changed pixels (colored white in the right images); particle #1 yields the smallest signal intensity difference, and #2 yields the second smallest. A moment with many #1-like particles outputs a smaller value of ΔF(t), averaged signal intensity difference, than that with many #2-like particles. Signal intensity difference sometimes underestimates the speed: e.g., particles #3 and #4 with different speeds produce the same area of signal intensity difference because both move to areas that exclude the previous areas; the middle particle of #5 is apparently vanished in the signal intensity difference images due to nearby particles. (PDF) [file pcbi.1013331.s001.pdf]

**S2 Fig**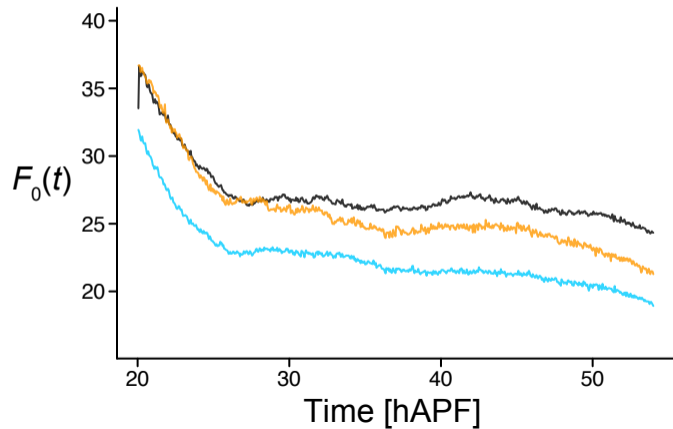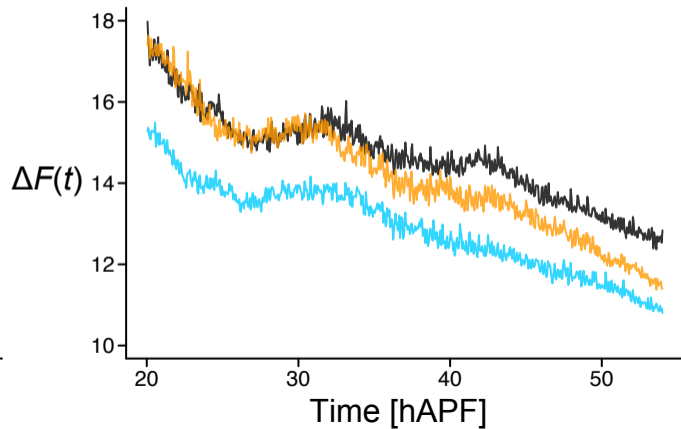

Supplement: S2 Fig — Left: average signal intensity of the original images (F0(t)), right: average signal intensity of the signal intensity difference images before normalization (ΔF(t)). The normalized signal intensity difference (ΔF(t)/F0(t)) is shown in Fig 1c with corresponding colors indicating three individuals. (PDF) [file pcbi.1013331.s002.pdf]

**S4 Fig**

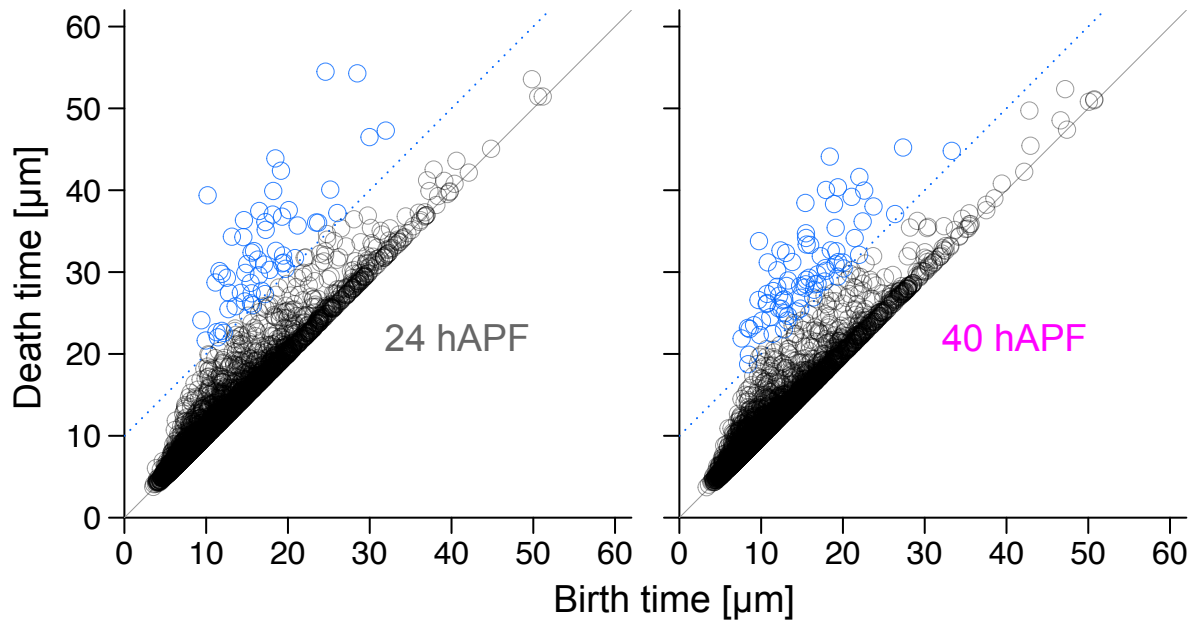

Supplement: S4 Fig — Left: 24 hAPF, right: 40 hAPF. The ‘ring-like’ structures based on persistent homology are annotated by blue circles with >10 μm life time, a threshold indicated by the blue dotted lines. The death time of the ‘ring-like’ structures is defined as the ring radius, whose histogram is shown in Fig 2c. (PDF) [file pcbi.1013331.s004.pdf]

**S5 Fig****a**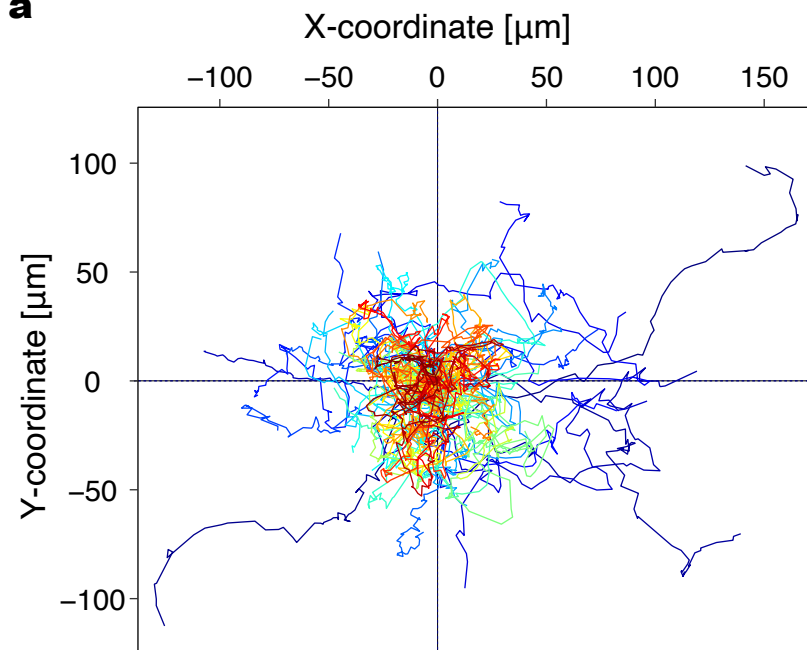**b**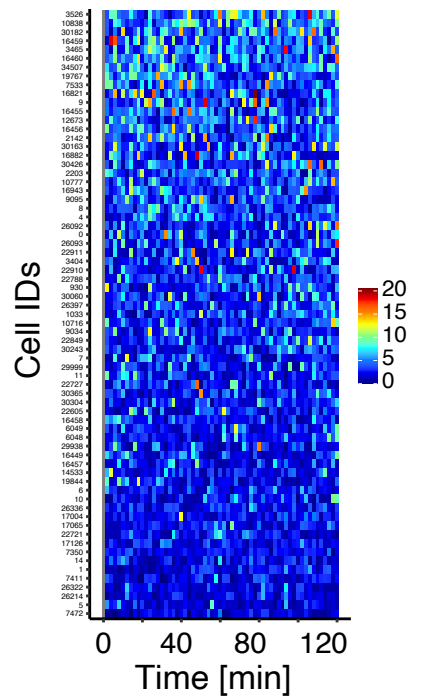**c**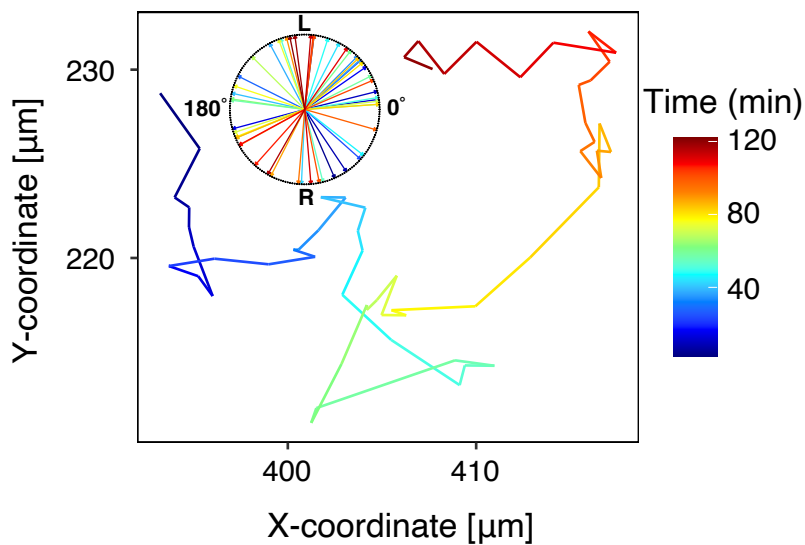**d**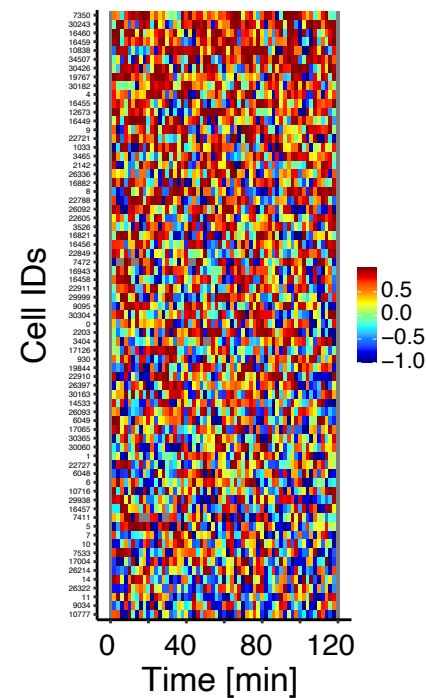

Supplement: S5 Fig — (a) Trajectories of all the analyzed hemocytes. The initial positions are aligned to the origin. The color code corresponds to the rank of total migratory trajectory length: red, short; blue, long. (b) Heatmap of instantaneous speed for individual hemocytes. Time series of instantaneous speed for individual cells are aligned horizontally. (c) A representative trajectory for a hemocyte. Color code indicates time. Circular plot indicates angular difference in moving orientation from a frame before at each time point. 0 deg to the right, 180 deg to the left, rightward to the top, and leftward to the bottom. (d) Heatmap of difference in moving orientation from a frame before at each time point for individual hemocytes. Time series of cosθi(n) (defined in Sect 2.1.4) for individual cells are aligned horizontally. (PDF) [file pcbi.1013331.s005.pdf]
