# Supplementary material for: Dual-purpose dynamics emerge from a heterogeneous cell population in Drosophila metamorphosis
Source: PLoS Comput Biol. 2025 Aug 28;21(8):e1013331. doi: 10.1371/journal.pcbi.1013331 (PMC12393715; doi:10.1371/journal.pcbi.1013331)
Supplement: S3 Fig — A ‘ring-like’ structure is recognizable in the left coordinate data but not in the right. This subjective assessment of ‘ring-likeness’ corresponds to the length of the life time—the subtraction of the birth time (roughly equivalent to half the average inter-point distance enclosing the ring) from the death time (roughly equivalent to the radius of the ring). In the actual coordinate data, a number of such structures can be detected throughout the field, generating multiple subsets of birth, death, and life times. (PDF) [file pcbi.1013331.s003.pdf]

S3 Fig

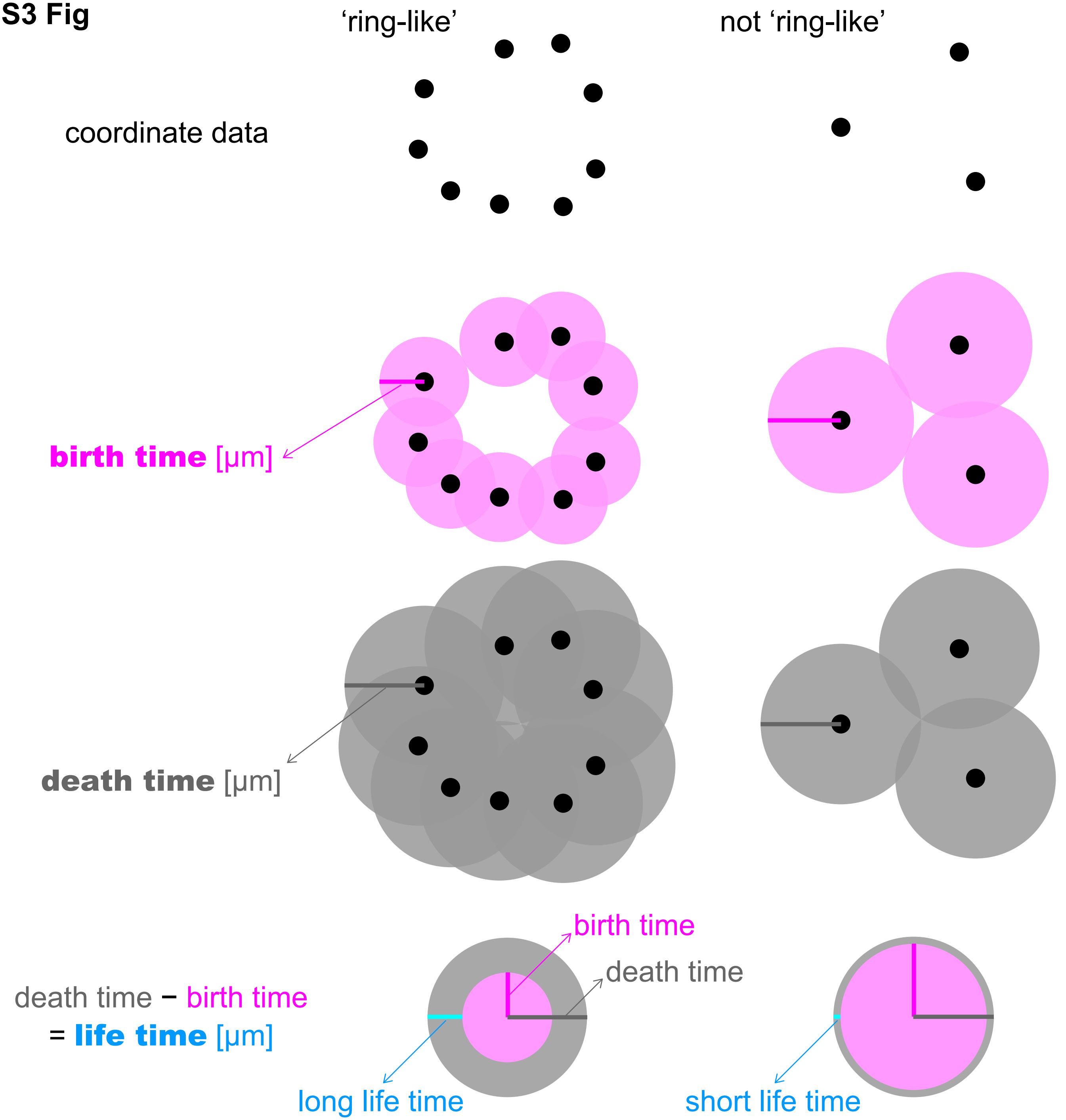

'ring-like'
not 'ring-like'
coordinate data
birth time [μm]
death time [μm]
birth time
death time
death time − birth time
= life time [μm]
long life time
short life time
