# Supplementary material for: Dual-purpose dynamics emerge from a heterogeneous cell population in Drosophila metamorphosis
Source: PLoS Comput Biol. 2025 Aug 28;21(8):e1013331. doi: 10.1371/journal.pcbi.1013331 (PMC12393715; doi:10.1371/journal.pcbi.1013331)
Supplement: S6 Fig — Snapshots are captured at 21 hAPF (step 64,800). Top left: no adhesion, aHF=aHH=aFF=0. Top right: adhesion between a hemocyte and a fat body cell, aHF=2.0 [nN] and aHH=aFF=0; these parameters are applied in the main simulations. Bottom left: adhesion between hemocytes, aHH=2.0 [nN] and aHF=aFF=0. Bottom right: adhesion between fat body cells, aFF=2.0 [nN] and aHF=aHH=0. Lines below the snapshots show the plot legend for the speed (top plot) and the horizontal variance (bottom plot). The plots are shown as in Fig 6d and 6e. In all the conditions, fat body cells start floating after 14 hAPF (shaded in the plots), and hemocytes are homogeneous in motility. See S6 Video for the simulation video. (PDF) [file pcbi.1013331.s006.pdf]

S6 Fig

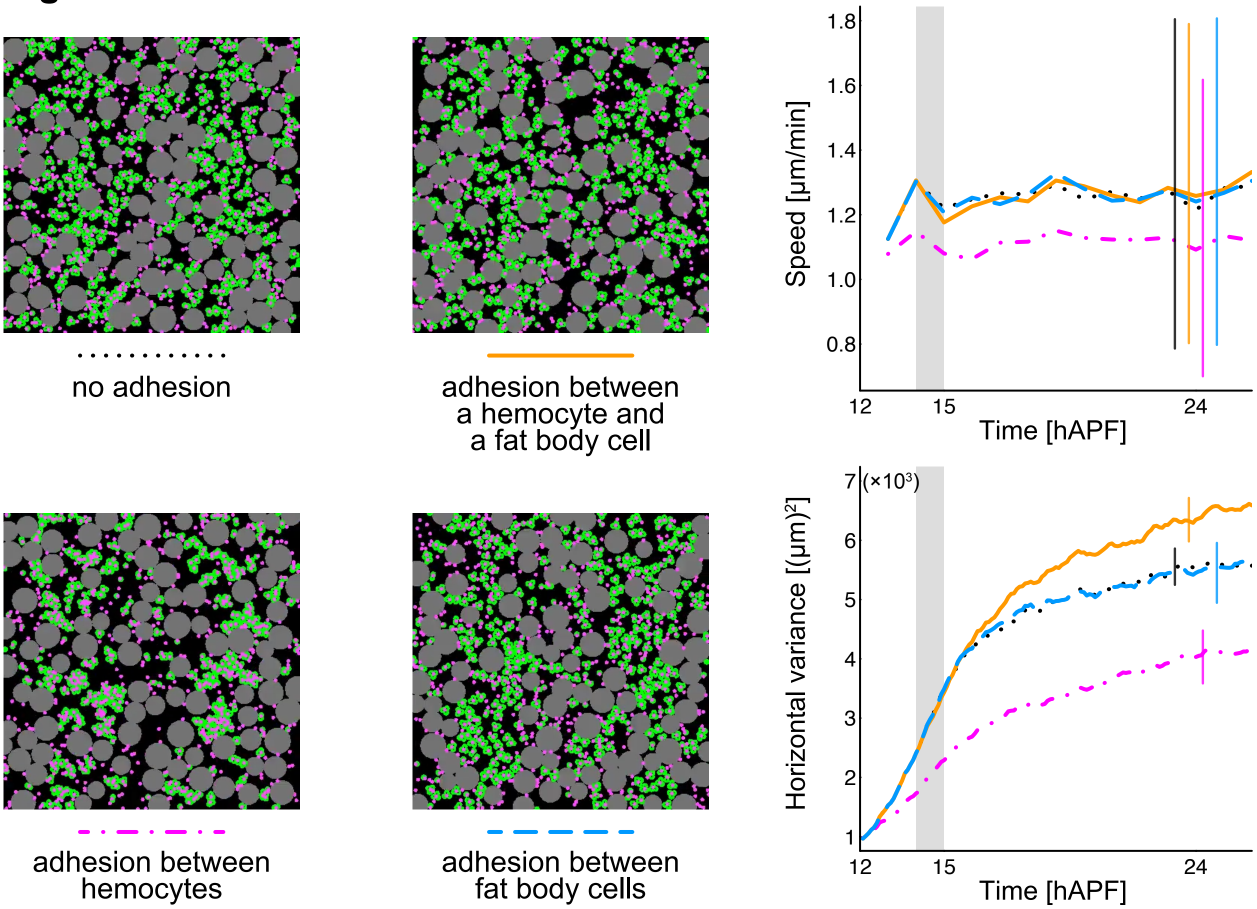
no adhesion
adhesion between a hemocyte and a fat body cell
adhesion between hemocytes
adhesion between fat body cells
Speed [µm/min]
1.8
1.6
1.4
1.2
1.0
0.8
12
15
24
Time [hAPF]
Horizontal variance [(µm)^2]
(×10^3)
7
6
5
4
3
2
1
12
15
24
Time [hAPF]
