# Supplementary material for: Dual-purpose dynamics emerge from a heterogeneous cell population in Drosophila metamorphosis
Source: PLoS Comput Biol. 2025 Aug 28;21(8):e1013331. doi: 10.1371/journal.pcbi.1013331 (PMC12393715; doi:10.1371/journal.pcbi.1013331)
Supplement: S7 Fig — For each operation, we perform simulations in which a parameter is gradually changed by a degree (explanatory variable on the x-axis) and obtain the percentage of the average speed of muscle units before the change to that after the change (Response variable on the y-axis). Each circle represents the measurement from a simulation trial. A series of simulations yields a regression line, by which we determine a parameter change that reduces the muscle unit speed by 80% (arrow). (PDF) [file pcbi.1013331.s007.pdf]

S7 Fig

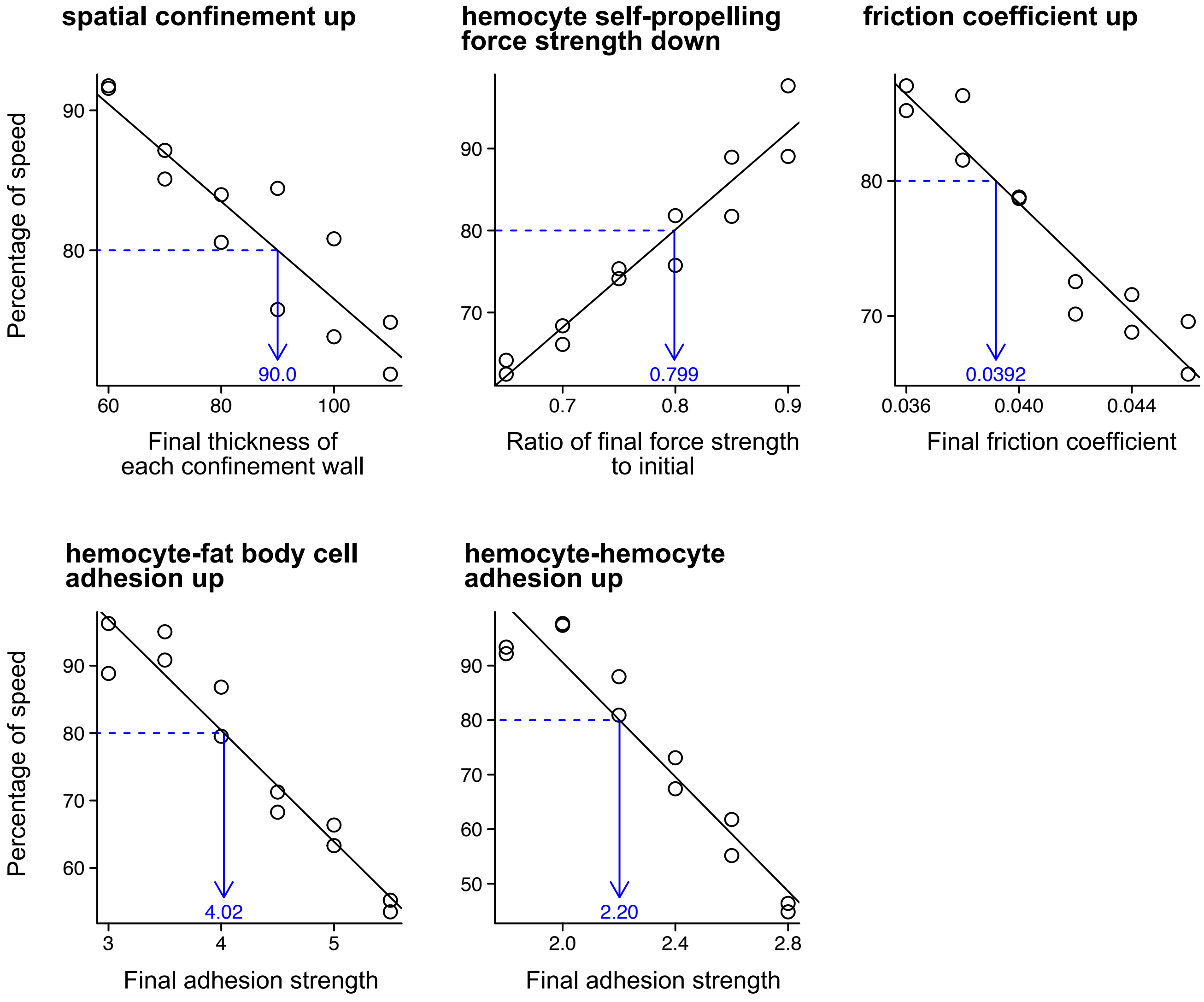
spatial confinement up
Percentage of speed
90
80
90.0
60
80
100
Final thickness of each confinement wall
hemocyte self-propelling force strength down
90
80
70
0.799
0.7
0.8
0.9
Ratio of final force strength to initial
friction coefficient up
80
70
0.0392
0.036
0.040
0.044
Final friction coefficient
hemocyte-fat body cell adhesion up
Percentage of speed
90
80
70
60
4.02
3
4
5
Final adhesion strength
hemocyte-hemocyte adhesion up
90
80
70
60
50
2.20
2.0
2.4
2.8
Final adhesion strength
