# Supplementary material for: Dual-purpose dynamics emerge from a heterogeneous cell population in Drosophila metamorphosis
Source: PLoS Comput Biol. 2025 Aug 28;21(8):e1013331. doi: 10.1371/journal.pcbi.1013331 (PMC12393715; doi:10.1371/journal.pcbi.1013331)
Supplement: Attachment — Submitted filename: Response.pdf [file pcbi.1013331.s016.pdf]

Dear Editorial Board of PLOS Computational Biology,

Thank you very much for taking your time to handle our manuscript entitled "Dual-purpose dynamics emerge from a heterogeneous cell population in *Drosophila* metamorphosis." We revised the manuscript according to the reviewers' valuable comments (colored blue).

Reviewers' comments:

#1-general) Reviewer #1: This paper reports an integrated in vivo imaging and agent-based modeling study on how cellular heterogeneity governs the collective behavior during muscle remodeling in Drosophila pupae during metamorphosis. The authors find the sarcolyte movement slows down over time and becomes more spatially uniform. They propose that macro-heterogeneity (across hemocytes and fat body cells) and micro-heterogeneity (within the same type, e.g., variability in hemocyte motility) contribute to both rapid dispersal and stable pattern formation, an interesting phenomenon they term "dual-purpose dynamics." Their agent-based model shows that delayed appearance of fat body cells facilitates early spread of sarcolytes followed by spatial stabilization, and variation in hemocyte motility allows dispersal while keeping sarcolytes locally stable. In addition, spatial confinement reproduces the slowing and pattern formation in the later stage.
Overall, the paper is very well written. In vivo imaging data are from multiple individuals and developmental time points with consistent trends and robustness. Appropriate statistical tests are used to quantify the changes in motion and spatial patterns. The agent-based model includes multiple cell types, differential adhesion, and motility heterogeneity (turning frequency), with biologically motivated rules and parameters.

RESPONSE: We thank reviewer #1 for the time to review our manuscript and the encouraging comments. We would like to respond point by point to the reviewer's suggestions below. The page and line numbers in this letter follow the manuscript that highlights our changes from the original submission (not the clean manuscript).

#1-1) The main complaint I have is the lack of model to experiment feedback – the model predictions do not inform more experiments to validate the discovery. The study would be significantly strengthened by an additional perturbation experiment, e.g., delay fat body emergence or modify hemocyte heterogeneity, to confirm causal relationship.

RESPONSE: We thank the reviewer's critical suggestion. As to the hemocyte heterogeneity, we found no available genes whose function is associated with heterogeneity in motion even nor with cell

subtypes. Thorough genetic screening should be performed to identify the molecular mechanisms that regulate the hemocyte heterogeneity. Even though this is interesting and important, this full-scale genetic approach is out of the focus of this work and we suppose that this is beyond the scope of publications on the journal.

Nevertheless, we sought to perform some genetic experiments based on a limited set of available knowledge. Given that the importance of the metamorphosis hormone, ecdysone, in regulation of hemocyte dynamics, we reasoned that we may be able to compromise the proper differentiation of hemocytes. However, inhibition of the ecdysone signaling in hemocytes affected the number of hemocyte making difficult to analyze the sole effect on sarcolyte dispersion and distribution but could impact on physiological effects (Figure i below).

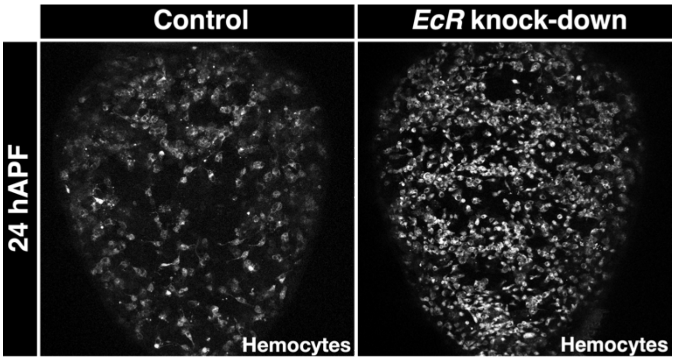

Figure i. Knocking-down of ecdysone receptor, EcR, specifically in hemocytes using the GAL4/UAS system with He-GAL4 as a driver caused increase in hemocyte number unexpectedly.

Removing fat body cells by inducing apoptosis most likely causes lethality or physiological disturbance given the multiple biological roles of the fat body (in fact, we tried once but the animal died at an early pupal stage).

Blocking MMP1 and MMP2 functions in fat body cells could efficiently delay the fat body disintegration. However, the strong inhibition of MMPs causes pupal lethality (Jia et al., Sci. Rep., 2014). Lethality caused by this genetic manipulation implies that the fat body impacts on physiology of the animal in a systemic manner. Therefore, we cannot rule out the possibility that interfering fat body dissociation by means of MMP loss of function would affects the physiology of hemocytes. Therefore, we refrained from discussing much about physics aspects from the experimental results but rather adhere to in silico analysis to evaluate physical properties of each heterogeneity. Since cell-cell adhesion is the most known downstream cellular activity in keeping fat body cells attach together (Jia et al., Sci. Rep., 2014), the only approach that we could come up with was to increase the cell-cell adhesion experimentally. Based on our research in an epithelium (Iijima et al., Nat. Commun., 2020), we sought to enhance the cell-cell adhesion by

misexpressing the extracellular domain of Tl gene product in fat body cells because the extracellular domain of Tl induces a strong cell-cell adhesion in both an epithelium and cultured non-adhesive cells. The animals were viable upon the misexpression of the extracellular domain of Tl. However, this genetic treatment did NOT interfere with fat body cell dispersion (Figure ii below), and therefore we were not able to assess the physics aspects of the presence/absence of fat body cells in vivo.

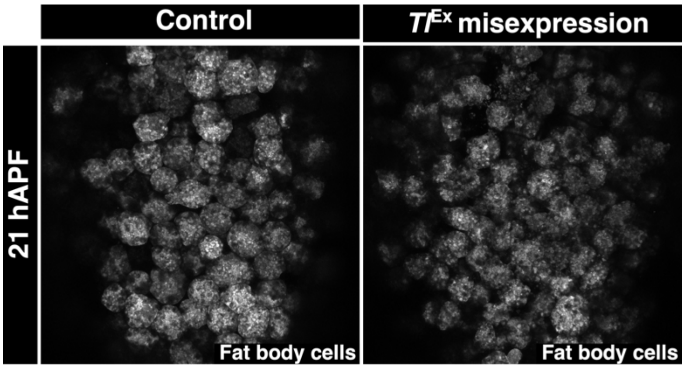

Figure ii. Forced expression of Tl extracellular domain specifically in fat body cells using the GAL4/UAS system with Lsp2-GAL4 as a driver did NOT affect disintegration of fat body cells.

For testing the predicted role of delayed fat body emergence on efficient spreading of sarcolytes, not the delay of the fat body cell dissociation but the precocious disintegration should be tested. However, this is challenging to evaluate with in vivo experiments. For example, a known manipulation to induce precocious disintegration of fat body cells causes pleiotropic effects on not only fat body cell dissociation but also hemocytes (Dolezal et al., PLoS Biol., 2005). Likewise, the over-expression of metalloproteinases, MMP1 or MMP2, causes precocious fat body disintegration but this genetic treatment results in early pupal lethality (Jia et al., Sci. Rep., 2014) preventing the analysis of sarcolyte dynamics in vivo.

Overall, the molecular mechanisms that regulate heterogeneity of hemocytes and fat body disintegration have not been sufficiently revealed to validate with reasonable in vivo experimental settings. The validation awaits thorough genetic studies in the future. Given the limitations mentioned above, we have added a brief discussion in Section 3.3 (Conclusion and outlook) as follows:

> "Further biological experiments are needed to validate the proposed benefits of macro- and micro-heterogeneity. Feedback from our in silico prediction to in vivo validation is, however, currently challenging due to limited knowledge of molecular mechanisms regulating hemocyte micro-heterogeneity and fat body disintegration, thus awaiting thorough genetic studies in the future." (Section 3.3, page 22, lines 497–501)

I also found the presentation of the data can be improved.

#1-2) Figure 1 (b): the visual distinction between static and moving sarcolytes in the difference images are not immediately intuitive. A color-coded image may be easier to understand than the grayscale differences.

RESPONSE: We agree with the suggestion. We have converted Fig 1b into color-coded images. Because the calculation of $\Delta F(t)/F_0(t)$ was performed in grayscale, we have added the following statement in the legend of Fig 1b: "While grayscale images are used for calculation, the displayed images are pseudo-colored to improve visibility: a large difference in bright yellow and a small difference in dark blue." We have kept S1 Video in grayscale because we consider that the animated signal intensity difference is intuitive enough.

#1-3) (c) is missing a legend.

RESPONSE: As far as we checked, (c) is not missing a legend. The legend of Fig 1 includes the followings: "(c) Normalized average signal intensity difference ($\Delta F(t)/F_0(t)$) in three individuals. The images in (a,b) are obtained from the individual of the black-colored plot."

#1-4) Can the signal intensity difference in Fig 1c be converted to speed and be compared with modeling result in e.g., Fig 8f?

RESPONSE: Yes. In Section 2.2.1, we intended to write how the signal intensity difference can be converted to speed. Because it might be difficult to understand, we have added an explicit statement as underlined below:

> "<u>If the signal intensity difference is linearly converted to the speed,</u> we can interpret that the speed of sarcolytes decreased by ~90% in 30–50 hAPF. However, signal intensity difference usually underestimates the original speed (S1 Fig). For example, when a sarcolyte has moved to a position at which another sarcolyte existed in the last frame, signal intensity around this position remains constantly high in some pixels, making signal intensity difference smaller than the extent of the actual displacement. Thus, the percentage in the actual speed is supposed to be lower than 90%." (Section 2.1.1, pages 6–7, lines 93–100)

The stabilization operations in the simulations were set up so that the speed of the muscle units

imitates the in vivo decrease in speed, which was roughly converted from the signal intensity difference. We thus suppose the modeling results are already comparable to Fig 1c. In Section 2.2.4, we have added an explicit statement as underlined below:

> "The degree of parameter changes was determined to resultantly decrease the speed of muscle units by 80% between before and after the stabilization operation (30 and 50 hAPF, respectively). Presuming that the decreasing rate of signal intensity difference from 32 to 50 hAPF in vivo, ~90%, would underestimate the original speed (S1 Fig; explained in the last of Section 2.1.1), we aimed a little lower criterion, 80%. We thus suppose that the decrease in speed in Fig 8f is comparable to the post-peak decrease in signal intensity difference in Fig 1c." (Section 2.2.4, page 16, lines 340–346)

We hope the above statement addresses the reviewer's concern.

#1-5) Figure 2 (a): overlaying sarcolyte coordinates from 6 individuals makes spatial trends difficult to discern. It might be a good idea to color-code individuals.

RESPONSE: The coordinate plots in Fig 2a are separated by individuals, not overlaid. The upper row indicates six individuals at 24 h, and the lower row indicates six individuals at 40 h. For example, the top left plot with 1258 sarcolytes is obtained from a single individual, not six. We revised the legend of Fig 2a to avoid such misunderstanding: "Upper: 24 hAPF, separately showing six individuals. Lower: 40 hAPF, separately showing six individuals, different ones from those in the upper row."

#1-6) Figure 4 (a): Cumulative displacement (panel a) is not the best format for comparing individual variability. Individual trajectory maps or vector fields would be more effective.

RESPONSE: We agree with the suggestion. In fact, we already showed the individual trajectories of all the analyzed hemocytes in the supplementary figure, S5 Fig (a). We have placed a simplified plot of S5 Fig (a) into the new Fig 4a, where we have extracted only the samples of those shown in the cumulative displacement plot (new Fig 4b) to clearly display each trajectory. We suppose that the cumulative displacement plot is also important to show that each hemocyte moved in a relatively constant pace, thus keeping it in the main figure. We also leave the original trajectory map of S5 Fig (a) as it was since it includes all available information.

#1-7) Figure 5. Adding a flowchart of model dynamics could help readers less familiar with agent-

based modeling.

RESPONSE: This is a great idea for broad readers. We have added flowcharts in Fig 5d and the corresponding sentence in Section 2.2.1 as below:

> "Under this agent-based model, the coordinates and state of each particle were updated every step according to contact-based and probabilistic decisions (Fig 5d)." (Section 2.2.1, page 12, lines 233–234)

Also in Section 4.4 (Modeling) in Methods, we have newly cited Fig 5d where needed (lines 588, 598, and 612).

#1-8) Figure 6. how many replicate simulations were done for each parameter set? Do we not expect error bars for speed and spatial variance measurements?

RESPONSE: Six replicate simulations were done for each condition. While we wrote it at the last of Section 4.5 (Simulation conditions), we have included it also in the legends of Figs 6–8 (pages 13, 15, and 17). Moreover, we have added error bars in Fig 6d,e, Fig 7d,e, and S6 Fig. To avoid visual complexity, the error bar for each condition is shown only at 24 h.

#1-9) Figuer 8. Not clear what error bars are, and if the differences are significant.

RESPONSE: The legend has the explanation of the error bars as: "Symbols and error bars in (g,h) show medians and interquartile ranges, respectively." For the significance, we have added the followings in the legend of Fig 8 (page 17):

> "The spatial confinement simulations show similar changes to the biological data, namely, increase in the rank average of the number of nearby muscle units (Brunner-Munzel test, $p < 0.001$) and decrease in the rank average of the ring radius (Brunner-Munzel test, $p = 0.0038$)."

We have also included the test name and p values in the main text (page 17, lines 352–354).

#1-10) Table 1: adding another column indicating if the model parameter values are estimated, measured, or fitted would be better.

RESPONSE: We agree with the importance to show the rationale of parameter values. Because Table 1 has a limited space to precisely explain it, we have decided to create a paragraph titled "Parameter setting" in Section 4.5 (Simulation conditions) to detail it as below:

> "**Parameter setting.** The radius parameters (muscle unit r_S, hemocyte r_H, and fat body cell r_F) were set according to biological knowledge [12,17] and our observations (Fig 3b). The friction coefficient μ and the self-propelling force strength of hemocytes $v_{H,i}$ were adjusted to yield a hemocyte speed of 1–3 μm/min, consistent with our experimental data (Fig 4b). The self-propelling force strength of fat body cells, which appeared to have a limited effect on the overall dynamics, was set to a small value. The turning probability p_i was tuned to reproduce the observed trajectories of hemocytes [14]. The repulsion-related parameter k was set to allow a partial overlap between cells given their deformable nature. Adhesion strength parameters (a_{HF}, a_{HH}, a_{FF}, and a_E) were tuned to reproduce a network-like arrangement of muscle units while avoiding unnatural clustering (S6 Fig)." (Section 4.5, page 28, lines 629–639)

#1-11) S1 Fig – I find it very confusing.

RESPONSE: We have omitted frame 3 and instead added an overlay image of frames 1 and 2 as an intermediate step. We hope this revision avoids a confusion.

#1-12) S3 Fig – standard TDA illustration, maybe no need to include

RESPONSE: Because we suppose that not every reader is familiar with TDA and the visual interpretation of the 'ring-like' structure is important in this study, we have decided to keep S3 Fig.

#2-general) Reviewer #2: The study presents computational results that complement their in vivo observations of muscle remodeling in Drosophila, offering insight into how cellular heterogeneity shapes collective behavior during development. The authors conceptualize the remodeling process as a dynamic interaction between migrating hemocytes, fat body cells, and decomposing muscle fragments (sarcolytes), and develop a mathematical model to probe the causal mechanisms underlying sarcolyte dynamics. They introduce computational tools to evaluate how macro-heterogeneity (across cell types) and micro-heterogeneity (within hemocyte behavior) contribute to the emergent spatial organization of sarcolytes, revealing a dual-purpose mechanism by which biological systems achieve

both rapid dispersal and patterned arrangement.

The figures, methods, and explanations collectively support the idea that the developed computational model is a reasonable representation of the empirical system. Overall, the references and detailed results are consistent and well-explained. The codes are included and well documented within the included material links.

Major Comments/Issues

-None

RESPONSE: We thank reviewer #2 for the time to review our manuscript and the encouraging comments. Below, we would like to respond to the reviewer's comment. The page and line numbers in this letter follow the manuscript that highlights our changes from the original submission (not the clean manuscript).

Minor Comments/ Issue

#2-1) 1. Grammar correction (Author Summary)

In the author summary the sentence: "We then used mathematical tools to find what affect the movement of muscle pieces."

The word affects should replace the word affect.

RESPONSE: We have corrected it (page 2).

#2-2) 2. Figure caption and terminology order (Section 2.1.1)

(line 72) Section 2.1.1 introduces hours after puparium formation (hAPF), the figures referenced in the draft appear before this introduction of hAPF.

While minor, it would improve readability to introduce hAPF before its first usage in the figures.

RESPONSE: We agree with the suggestion. We have added the non-abbreviated term in the legend of Fig 1 (page 5).

#2-3) 3. Definition of "network-like arrangement" (Section 2.1.2)

The definition of a somewhat network like arrangement is not clear from the provided images, either providing a colored panel highlighting this in the images and/or a definition such as network-like arrangement typically refers to a spatial organization of cells or cellular structures that resembles a mesh, web, or interconnected lattice.

Even if the term is familiar within the field, a definition or citation would strengthen the text.

RESPONSE: We agree with the suggestion. In Fig 1, we have added an inset where colored curves highlight a possible interpretation of the 'network-like' arrangement. Moreover, at the beginning of Section 2.1.2, we have explicitly defined the 'network-like' arrangement as underlined below:

> "Visual inspection gave us the impression that sarcolytes are arranged in a somewhat network-like arrangement (Fig 1a, right inset), which is a mesh- or web-like structure where multiple particles comprise its nodes and links." (Section 2.1.2, page 7, lines 105–106)

#2-4) 4. Force representation clarification (Line 573)

The force repulsion term:

(-k(ri+rj-Xj(t)-Xi(t)|)

This gives the repulsion terms between different particles, this might need more expansion in the details of the justification of why add the two radii sizes in the repulsion representation. Is this meant to be an approximation given along with the unit vector between the two coordinate points of the circumference area of one particle directed at another?

The primary focus for the force seems to be the aij term, but the overall strength of the work would benefit if the repulsive terms were elaborated upon.

RESPONSE: The expression $r_i + r_j - |\mathbf{x}_j(t) - \mathbf{x}_i(t)|$ represents the overlapping length of two particles, as shown in the drawing below:

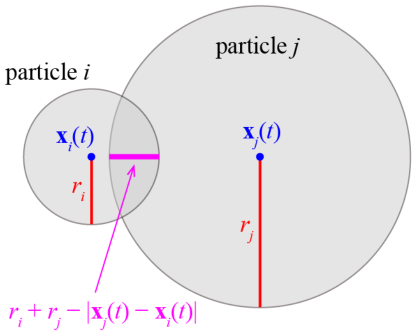

We included the radii of two particles (i and j) to represent the contact of particles, whose radii may differ by case. For example, in the interaction between a sarcolyte with $r_i = 6$ and a fat body

cell with $r_j = 23$, the expression $r_i + r_j - |\mathbf{x}_j(t) - \mathbf{x}_i(t)|$ becomes 0 when the distance $|\mathbf{x}_j(t) - \mathbf{x}_i(t)|$ equals 29 µm (when the two circles are externally tangent) and becomes positive when the distance is shorter than 29 µm (when the two circles are overlapped). The longer the overlapping length is, the stronger the repulsion is due to a larger excluded volume effect. The overlapping case results in a negative value of $- k (r_i + r_j - |\mathbf{x}_j(t) - \mathbf{x}_i(t)|)$, and thus particle i moves away from particle j by the repulsion since the unit vector is directed from i to j. After Eq 4 in Section 4.4 (Modeling), we have added a detailed explanation for the repulsion as below:

> "The expression $r_i + r_j - |\mathbf{x}_j(t) - \mathbf{x}_i(t)|$ represents the overlapping length of particles i and j. Due to multiplication by a negative coefficient $- k$, the more the two particles overlap, the stronger the repulsion between them." (Section 4.4, page 26, lines 603–605)

We hope that we precisely understand the reviewer's concern.

#2-5) 5. Radius growth clarification (Page 27, lines 617–620)

The statement about fat body cell growth isn't entirely clear. At a growth rate of 0.45 µm per minute, the radius would increase by 27 µm over one hour, which exceeds the stated average radius of 23 µm.

Consider rephrasing this section for clarity

RESPONSE: This comment is right. The growth rate should be 23/60 = 0.383 µm per minute. Although the simulation code was correct, the manuscript writing was wrong and thus has been revised. We thank the reviewer for the very careful calculation.

#2-6) 6. Follow-up on fat body cell growth modeling

Building on Comment 5:

Is there any plan to explore more biologically motivated growth dynamics, such as varying the fat body cell radius based on spatial constraints, relative location, or developmental timing, rather than applying a constant rate of increase?

Incorporating such variation might better reflect biological heterogeneity and could yield further insights into future work.

RESPONSE: We thank the reviewer for the suggestion. Indeed, considering other forms of biological heterogeneity would highlight a more comprehensive mechanism. As a future work, we have added the following discussion at the end of Section 3.2 (Advantage of micro-heterogeneity):

"Micro-heterogeneity in other cell types, such as fat body cells, may also provide advantages. In our simulations, round-shaped fat body cells floated simultaneously at a uniform rate throughout the field. However, further biological observations of the fat body dynamics could reveal micro-heterogeneity in dissociation and floating processes as well as cell morphology and motility. For example, a previous study reported the migratory properties of fat body cells during wound healing [17], in which we can recognize considerable variations in speed and meandering measurements. Moreover, such variations may be influenced by internal environmental factors, including spatial constraints imposed by the pupal structure. Incorporating additional variability into simulations could uncover further roles of cellular micro-heterogeneity." (Section 3.2, page 21, lines 471–480)

We sincerely appreciate the editor's and reviewers' time and thoughtful feedback, which have improved our manuscript.

Sincerely yours,

Daiki Wakita, Satoshi Yamaji, Daiki Umetsu, and Takeshi Kano

Correspondence:

Takeshi Kano, M.D., Ph.D.

Professor

School of Systems Information Science,

Future University Hakodate

116-2 Kamedanakano-cho, Hakodate, Hokkaido 041-8655, Japan

Tel: +81-138-34-6425

E-mail: tkano@fun.ac.jp

Co-correspondence:

Daiki Umetsu, Ph.D.

Lecturer

Graduate School of Science,

The University of Osaka

1-1, Machikaneyama-cho, Toyonaka, Osaka 560-0043, Japan

E-mail: daiki.umetsu.sci@osaka-u.ac.jp
